# The effect of the COVID-19 pandemic on life expectancy in the USA: An application of hybrid life expectancy

**DOI:** 10.1093/biomethods/bpad025

**Published:** 2023-10-18

**Authors:** Warren C Sanderson, Sergei Scherbov

**Affiliations:** Department of Economics, Stony Brook University; Stony Brook, NY 11794, United States; Center of Demographic Research, Baruch College, New York, NY 10010, United States; Population and Just Societies Program, International Institute for Applied Systems Analysis, Laxenburg A-2361, Austria; Population and Just Societies Program, International Institute for Applied Systems Analysis, Laxenburg A-2361, Austria; College of Population Studies, Chulalongkorn University, Bangkok 10330, Thailand

**Keywords:** hybrid life expectancy, COVID-19 pandemic, life expectancy, pandemic duration

## Abstract

Pandemics are, by definition, temporary intervals of substantially increased mortality rates experienced across a wide geographic area. One way of assessing the magnitude of the COVID-19 pandemic in the USA has been to compute the differences in life expectancy at birth during a pandemic year and the year before the pandemic. Such comparisons are misleading because they do not account for the duration of the pandemic. The computation of life expectancy in 2019 assumes that people spend their entire lives experiencing prepandemic mortality rates. The computation of life expectancy in 2021 assumes that people live their entire lives in a permanent pandemic. However, people do not live their entire lives experiencing the elevated mortality rates of 2021. This article introduces a method for calculating life expectancy that reflects the experience of people enduring pandemic-level mortality rates for fixed durations. We call the new quantity *hybrid life expectancy* because it integrates both pandemic and prepandemic mortality rates. The difference in life expectancy at birth in the USA in 2019 with and without a 3-year-long pandemic is 0.01 years. This is because mortality rates at ages 0, 1, and 2 in the pandemic were essentially unchanged from their prepandemic levels. Life expectancy at age 65 incorporating a 3-year pandemic is 0.18 years lower than life expectancy would have been without it. Reductions in life expectancy due to the COVID-19 pandemic using hybrid life expectancy are dramatically lower than differences in life expectancy that do not take the duration of the pandemic into account.

## Introduction

According to the Human Mortality Database (HMD), life expectancy at birth (both sexes) in the USA fell from 78.99 years in 2019 to 77.07 years in 2020 and then further to 76.43 years in 2021 [1]. This decline has been widely discussed in the media [2–4]. A press release from the CDC’s National Center for Health Statistics (NCHS) states that the life expectancy decline from 2019 to 2021 “was the biggest two-year decline in life expectancy since 1921-1923.” [5]. A website of America’s Public Broadcasting System (PBS) quotes a social epidemiologist referring to the life expectancy decline from 2019 to 2020 as saying “A loss of two years seems limited, but that’s rolling back decades and decades of progress.” [6]. Statements like these shape policy-makers understanding of the magnitude of the COVID-19 pandemic and they affect how people perceive and react to pandemic-related policies.

Demographers understand that changes in life expectancy between a prepandemic year and a pandemic year are biased measures of the pandemic’s effect [7, 8]. The calculation of life expectancy assumes a group closed to migration and a set of mortality rates. Given those mortality rates, life expectancy at a given age is the average number of years still to be lived by people who get to that age. When the mortality rates are those experienced by a cohort over their lifetime, the resulting life expectancy is called cohort life expectancy. Cohort life expectancy is not often calculated. A cohort life expectancy of people born in 2023 could only be computed sometime after 2123 because that is when all the necessary mortality rates would have been observed.

More frequently, period life expectancy is computed. Period life expectancy uses the mortality rates from a given period not those experienced by any cohort. For example, period life expectancy at birth in 2019 in the HMD is computed using the mortality rates of people from birth onward in 2019. Period life expectancy in 2021, a year of relatively high mortality rates, is computed assuming that people experience those high mortality rates for their entire lives. In other words, it assumes that people live their lives in a permanent pandemic. However, pandemics, by definition, are time-limited.

In this article, we present a new form of life expectancy that we call *hybrid life expectancy*. Hybrid life expectancy borrows a feature of period life expectancy in that it uses period mortality rates for a nonpandemic year. Hybrid life expectancy also borrows a feature of cohort life expectancy in that it follows people over time for the duration of the pandemic.

The SARS-CoV-2 virus is a cause of over 1 million deaths in the USA: 385 666 in 2020, 463 263 in 2021, 246 161 in 2022, and probably around 100 000 in 2023 [9]. There is no formal definition of when a pandemic ends in a particular place, but the public health emergency in the USA lasted for about 3 years, from 13 March 2020 to 11 May 2023.

## Materials and methods

Our data come from the US life tables (both sexes) in the HMD [1]. We use data from 2019, the year before the beginning of the COVID-19 pandemic, and 2021, the year during the pandemic with the highest number of COVID-19 deaths.

Life expectancy typically refers to populations all of whose members are assumed to be born on 1 January of a specific year [10]. Period life expectancy at age *a* in any year, *t*, is computed from the age-specific mortality rates in that year. It is the ratio of person-years lived from age *a* to the end of life, *T*(*a*, *t*), to the number of people who get to age *a*, *l*(*a*, *t*). In other words, it is the average number of years people who get to age *a* would live.


(1) $$\mathrm{ex}\left(a,t\right)=\frac{T\left(a,t\right)}{l\left(a,t\right)},$$

where ex(*a*, *t*) is the period life expectancy of people of age *a*, given the mortality rates of year *t*. Table 1 shows an excerpt from the HMD for the USA (both sexes) in 2019, a prepandemic year, and 2021, the worst year of the pandemic.

**Table 1.** Excerpt from the HMD: USA, 2019 and 2021, both sexes

Age	*l*(*a*, *t*)	*T*(*a*, *t*)	*e*(*a*, *t*)
2019 (prepandemic year)			
0	100 000	7 896 609	78.97
…			
65	83 925	1 653 780	19.71
66	82 861	1 570 387	18.95
67	81 734	1 488 090	18.21
68	80 548	1 406 949	17.47
…			
2021 (pandemic year)			
0	100 000	7 642 591	76.43
…			
65	79 550	1 471 884	18.50
66	78 289	1 392 965	17.79
67	76 978	1 315 331	17.09
68	75 608	1 239 038	16.39
…			

*Notes*: Setting *l*(*0*, *t*) to 100 000 is a standard demographic assumption. The value of *l*(*0*, *t*) does not influence life expectancy.

*Source*: [1], downloaded 18 September 2023.

Hybrid life expectancy at age *a* is also the ratio of the number of person-years people who get to age *a* can expect to live. Conceptually, this is the same as with period life expectancy. The difference with period life expectancy is that hybrid life expectancy assumes that people experience the elevated pandemic mortality rates only between ages *a* and *a + d*, where *d* is the duration of the pandemic.


(2) $$\mathrm{exh}\left(a,n,p,d\right)=\frac{T\left(a,n,p,d\right)}{l\left(a,n\right)},$$

where exh(*a*, *n*, *p*, *d*) is the hybrid life expectancy of people who begin to experience a pandemic of duration *d* at age *a*, *n* is a prepandemic year, *p* is a pandemic year, and *T*(*a*, *n*, *p*, *d*) is the number of person-years lived by people of age *a*, who experience pandemic-level mortality rates for a period from age *a* to *a* + *d*, but otherwise experience the mortality rates of the prepandemic year.

The numerator of the ratio can be expressed as:


(3) $$T\left(a,n,p,d\right)=l\left(a,n\right)*\left[\frac{T\left(a,p\right)-T\left(a+d,p\right)}{l\left(a,p\right)}\right]+l\left(a,n\right)*\frac{l\left(a+d,p\right)}{l\left(a,p\right)}*\frac{T\left(a+d,n\right)}{l\left(a+d,n\right)}.$$

Note that when there is no pandemic (*n *=* p* and *d *=* *0),


$$T\left(a,n,n,0\right)=\left[0\right]+T\left(a,n\right).$$

The number of person-years people of age *a* live can be divided into two parts. The first is the number of person-years lived during the pandemic and the second is the number of person-years lived after the pandemic is over. The term $\left[\frac{T\left(a,p\right)-T\left(a+d,p\right)}{l\left(a,p\right)}\right]$ is the number of person-years lived during the pandemic from age *a* to age *a + d* per person who got to age *a* during the pandemic. This is multiplied by the number of people who would get to age *a* in the prepandemic year. The number of person-years lived after the pandemic is the average number of person-years that people lived from the end of the pandemic onward multiplied by the number of people who survived to the end of the pandemic.

Therefore,


(4) $$\mathrm{exh}\left(a,n,p,d\right)=\left[\frac{T\left(a,p\right)-T\left(a+d,p\right)}{l\left(a,p\right)}\right]+\frac{l\left(a+d,p\right)}{l\left(a,p\right)}*\frac{T\left(a+d,n\right)}{l\left(a+d,n\right)}.$$

When there is no pandemic (*n *=* p* and *d *=* *0),


$$\mathrm{exh}\left(a,n,n,0\right)=\left[\frac{T\left(a,n\right)}{l\left(a,n\right)}\right]=\mathrm{ex}\left(a,n\right),$$

which is life expectancy at age *a* in the prepandemic year.

So period life expectancy, ex(*a*, *n*), is a special case of hybrid life expectancy, exh(*a*, *n*, *p*, *d*).

Using Equation (4) and the numbers in Table 1, it is straightforward to compute hybrid life expectancy. For example, the hybrid life expectancy of people experiencing a 3-year pandemic beginning at age 65 has two terms. The first is the number of person-years lived during the pandemic per person who would have reached age *a* given pandemic mortality rates. It is 2.93 years.


$$2.93=\frac{1471884-1239038}{79550}.$$

The second term is 16.60, the number of person-years per person lived by those who survived the pandemic.


$$16.60=\frac{75608}{79550}*\frac{1406949}{80548\;}.$$

The sum of the two terms is 19.53. Period life expectancy at age 65 in 2019 was 19.71. The reduction of life expectancy due to a 3-year pandemic would be 0.18 years. We cannot compare this reduction to an observed one because data for 2022 do not exist at the time of writing. We can compare the observed reduction in life expectancy at age 65 between 2019 and 2021 with the one measured using hybrid life expectancy. Life expectancy at age 65 in 2021 was 18.50 years, 1.21 years lower than it was in 2019. Using hybrid life expectancy, we see that the reduction in life expectancy due to a 2-year pandemic is 0.12 years, one-tenth of the life expectancy change calculated ignoring the duration of the pandemic. The main reason why the reduction in life expectancy is so small when measured using hybrid life expectancy is that the proportion of 65-year-olds who survive to age 67 was not so different in the 2019 and 2021 life tables. In 2019, it was 0.974 and in 2021, it was 0.968.

## Results

Applying Equation (4) to the figures in Table 1 from the HMD life tables for 2019 and 2021, we obtain the results in Table 2. If there were no pandemic and people experienced the mortality rates observed in 2019, they would have the life expectancy shown in the row labeled “Life Expectancy, 2019 mortality rates.” If instead of experiencing the 2019 mortality rates for every year of their lives, people experienced the elevated mortality rates of 2021 for 3 years (2020–2022), their life expectancy would be that shown in the row labeled “Life Expectancy, 2019 mortality rates, Pandemic Duration = 3 years.” The reductions in life expectancy range from 0.01 years at age 0 to 0.20 years at age 80.

**Table 2.** Life expectancy reductions due a 3-year long pandemic (2020–2022), USA, both sexes

	Age			
	0	20	65	80
Life expectancy, 2019 mortality rates	78.97	59.72	19.71	9.45
Life expectancy, 2019 mortality rates, pandemic duration = 3 years	78.96	59.68	19.53	9.25
Life expectancy reduction, pandemic duration = 3 years	0.01	0.04	0.18	0.20

*Note*: The life expectancy reductions in this table were calculated using Equation (4) and data from the HMD [1].

## Discussion

The life expectancy reductions shown in Table 2 are small compared with reductions computed from life expectancy differences that do not account for the duration of the pandemic. In Table 2, we show that the hybrid life expectancy of 65-year-olds in 2019 experiencing a 3-year pandemic would be 19.53. This number does not indicate a life expectancy regression of decades. It only means that the 2019 life expectancy of 65-year-olds accounting for a 3-year pandemic would be essentially the same as that observed in 2017 (19.52 years) [1]. Life expectancy reductions assuming a 4- or a 5-year-long pandemic are only marginally larger than those shown in Table 2.

Although the life expectancy reductions shown in Table 2 are comparatively small, they are, by design, overestimates. This is for two reasons. First, for each pandemic year, we used mortality rates from 2021, the year with the highest number of COVID-19 deaths. Life tables for the USA in the HMD are not yet available for 2022, but if they were and we used mortality rates for 2020, 2021, and 2022 separately we would likely have obtained a smaller life expectancy reduction. Mortality rates for 2020 were generally lower than for 2021 and mortality rates for 2022 are likely to be even lower. Second, deaths from COVID-19 were likely concentrated among those with the most serious preexisting medical problems. Survivors of the pandemic would be expected to be relatively healthy and have a higher life expectancy. However, long-COVID can exacerbate preexisting medical conditions or produce new ones [11]. Vaccination is an important reason for the decline in deaths from COVID-19. It also reduces the prevalence of long-COVID [12]. Long-COVID affects the health of individuals, but the extent to which it would affect long-term survival is currently unknown, so we cannot take it into account here.

It is inappropriate to assess the severity of a pandemic based on life expectancy differences between a pandemic and a prepandemic year. One figure would result from comparing 2020 and 2019, another from comparing 2021 and 2019, and another from comparing 2022 and 2019. None of these would reflect the cumulative effect of the pandemic because none of them would take the duration of the pandemic into account.

In this article, we present a simple formula for translating publicly available data into hybrid life expectancy, a new form of life expectancy that accounts for the duration of pandemics. Observations based on hybrid life expectancy can aid in policy formation by producing new consistent cross-country information on life expectancy reductions as well as new data on differentials across socio-economic groups.

## Data Availability

All data used in this article and open source and come from the HMD [1]. Computer code availability: The results in Table 2 were computed using Equation (4) and the data in Table 1. The computation was done using Excel. No computer code was written for the article.
